# Comparison of Test Your Memory and Montreal Cognitive Assessment Measures in Parkinson's Disease

**DOI:** 10.1155/2016/1012847

**Published:** 2016-07-05

**Authors:** Emily J. Henderson, Howard Chu, Daisy M. Gaunt, Alan L. Whone, Yoav Ben-Shlomo, Veronica Lyell

**Affiliations:** ^1^School of Social and Community Medicine, University of Bristol, Canynge Hall, 39 Whatley Road, Bristol BS8 2PS, UK; ^2^Taunton and Somerset NHS Foundation Trust, Musgrove Park Hospital, Parkfield Drive, Taunton, Somerset TA1 5DA, UK; ^3^Bristol Randomised Trials Collaboration, School of Social and Community Medicine, Canynge Hall, 39 Whatley Road, Bristol BS8 2PS, UK; ^4^Movement Disorders Group, Bristol Brain Centre, Southmead Hospital, Bristol BS10 5NB, UK; ^5^Royal United Hospital NHS Foundation Trust, Combe Park, Bath BA1 3NG, UK

## Abstract

*Background.* MoCA is widely used in Parkinson's disease (PD) to assess cognition. The Test Your Memory (TYM) test is a cognitive screening tool that is self-administered.* Objectives.* We sought to determine (a) the optimal value of TYM to discriminate between PD patients with and without cognitive deficits on MoCA testing, (b) equivalent MoCA and TYM scores, and (c) interrater reliability in TYM testing.* Methods.* We assessed the discriminant ability of TYM and the equivalence between TYM and MoCA scores and measured the interrater reliability between three raters.* Results.* Of the 135 subjects that completed both tests, 55% had cognitive impairment according to MoCA. A MoCA score of 25 was equivalent to a TYM score of 43-44. The area under the receiver operator characteristic (ROC) curve for TYM to differentiate between PD-normal and PD-cognitive impairment was 0.82 (95% CI 0.75 to 0.89). The optimal cutoff to distinguish PD-cognitive impairment from PD-normal was ≤45 (sensitivity 90.5%, specificity 59%) thereby correctly classifying 76.3% of patients with PD-cognitive impairment. Interrater agreement was high (0.97) and TYM was completed in under 7 minutes (interquartile range 5.33 to 8.52 minutes).* Conclusions.* The TYM test is a useful and less resource intensive screening test for cognitive deficits in PD.

## 1. Introduction

Cognitive impairment in Parkinson's disease (PD) is common and associated with functional impairment and poor quality of life [1, 2]. The spectrum of dysfunction ranges from executive dysfunction to mild cognitive impairment (MCI) seen even in early PD (PD-MCI), through to Parkinson's disease dementia (PDD). PDD has a cumulative incidence of 80% and is associated with significant morbidity, mortality, and carer stress [1, 3–5]. As the presence of MCI is associated with the development of dementia [6–8] and cognitive deficits impact quality of life [9], accurate identification of those with early cognitive changes is important to facilitate early planning, support, and intervention.

The Montreal Cognitive Test (MoCA) is increasingly used to screen for cognitive deficits, largely replacing the less sensitive Mini Mental State Examination (MMSE) [10–12]. MoCA takes 10–15 minutes to administer and assesses seven cognitive domains: visuospatial/executive (5 points), naming (3 points), attention (6 points), language (3 points), abstraction (2 points), memory (5 points), and orientation (6 points), yielding a total possible score of 30. One point is added if the individual has ≤12 years of education. Two studies have examined MoCA as a screening test for cognitive impairment in PD. To identify possible PD-MCI with >80% sensitivity, MoCA cutoff scores of 26/27 [10] or <26/30 [11] have been advocated. The Movement Disorder Society (MDS) Task Force for the diagnosis of PD-MCI (level 1 criteria) supports the use of MoCA to demonstrate global cognitive deficits in a clinical setting [13].

The Test Your Memory (TYM) (available from http://www.tymtest.com/) scale is a self-administered test that is validated in Alzheimer's disease (AD) and has been used in different regions (including in other languages) and clinical settings [14–16]. TYM's distinct advantage is that it reduces demands on clinical time as it can be supervised by nonclinical staff. TYM tests the same domains as MoCA: orientation (10 points), ability to copy a sentence (2 points), semantic knowledge (3 points), calculation (4 points), verbal fluency (4 points), similarities (4 points), naming (5 points), visuospatial abilities (2 tasks, total 7 points), and recall of a copied sentence (6 points). Ability to complete the test without assistance is scored (executive function, 5 points), yielding a total possible score of 50. For both tests, a higher score indicates better performance. With constraints on clinical time, TYM may represent a helpful additional or alternative tool to screen for cognitive deficits in PD.

This substudy sought to determine the ability of TYM to detect cognitive deficits in PD, determine equivalence between TYM and MoCA scores in PD, and assess interrater reliability of TYM scoring.

## 2. Materials and Methods

### 2.1. Study Population

We undertook a diagnostic test study nested within the ReSPonD trial, a double blind randomised controlled trial of Rivastigmine versus placebo to stabilise gait in people with PD [17]. Patients were invited to attend a screening clinic appointment if they appeared to meet the eligibility criteria for the ReSPonD trial. We sought to identify participants with idiopathic Parkinson's who did not have established dementia, were not treated with cholinesterase inhibitors, were able to walk 18 m, and had been stable on PD medication for 2 weeks. Patients were excluded if they had neurological, visual, or orthopaedic problems that interfered with balance or gait or were non-English speaking (cognitive tests were performed in English). Potential participants were identified from community and hospital settings, through registers and publicity campaigns.

Interested participants were sent an information pack and, if interested, had their eligibility checked by telephone. They were then invited for a face-to-face assessment when they completed the MoCA as part of the screening protocol. All patients at this visit were invited to participate in the TYM study regardless of their subsequent involvement in the ReSPonD trial. We excluded patients from the drug trial who had overt PD-dementia, the diagnosis of which was operationalised using the Movement Disorder Society Task Force definition of decreased cognition of sufficient severity to impair daily life [18]. Patients with a low MoCA score without clinically overt dementia (on global clinical assessment) were not excluded. Ethical approval was granted from the South West Central Bristol Research Ethics Committee and written informed consent was obtained from participants.

### 2.2. Procedures

Basic demographic information was obtained for all participants who were assessed in a clinically defined “on” medication state. More in-depth demographic and clinical information was gathered for the participants who subsequently enrolled in the RCT. MoCA and TYM were performed by trained research staff in a variable but nonrandomised order. The MoCA was completed by a registrar in geriatric medicine or trained research nurse, both of whom supervised and timed the TYM tests. All TYM tests were scored by a medical student (HC). To assess interrater reliability, 30% (*n* = 40/135) were additionally scored by two other individuals, a consultant geriatrician (VL) and a research assistant with no clinical experience, both of whom were provided with the published marking instructions only.

### 2.3. Statistical Analysis

Baseline data are described as mean ± SD if normally distributed or as median interquartile range (25th percentile, 75th percentile) if skewed. As TYM and MoCA are scored on different scales, equipercentile equating with log-linear smoothing [19] was undertaken using the “equate” package developed for “*R*”. Equally ranked percentiles are considered equivalent for the two scores and a conversion table is produced.

We used published screening criteria to classify participants as “PD-normal” (MoCA score 26–30), “PD-MCI” (MoCA score 21–25), and “PDD” (MoCA < 21) [11]. We then grouped PD-MCI and PDD into one group (“PD-cognitive impairment”). A receiver operating characteristic (ROC) was plotted and the area under the curve (AUC) was calculated to determine the ability of worsening TYM score to discriminate between PD-normal (MoCA score 26–30) versus PD-cognitive impairment (MoCA score ≤ 25). The optimal TYM screening cutoff was calculated by maximising Youden's *J* statistic [20] which gives equal weighting to sensitivity and specificity.

To assess reliability of TYM, we calculated the intraclass correlation coefficient (ICC) for interrater agreement using a two-way random-effects model assuming that the raters were randomly drawn from the population. The ICC is the ratio of intersubject variability to the total variability, defined as the sum of the intersubject variability, the between rater variability, and error variability. An ICC greater than 0.80 is regarded as indicative of high reliability [21]. Absolute difference between raters on TYM was calculated using a gold standard rater (VL) and subtracting the individual scores of the other two raters (i.e., VL TYM score minus other rater TYM scores). Statistical analysis was performed using Stata version 13.1 and “*R*” [22].

## 3. Results

### 3.1. Screening and Demographic Characteristics

**Figure 1:**
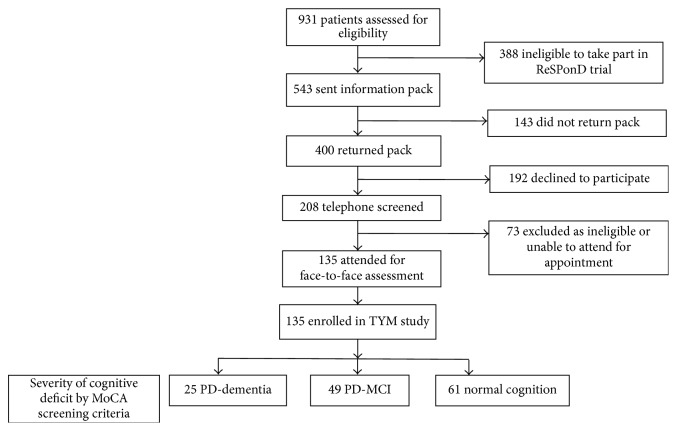
Patient flow.

Overall, 931 patients were screened for potential inclusion in the study. Of these, 500 (54%) did not meet the trial eligibility criteria, regardless of whether they wished to participate. Of the remaining 301 who were not enrolled and were potentially eligible, 143 did not reply to the initial invitation to attend and 158 declined to participate. Therefore, 135 attended for face-to-face screening, of whom 130 went on to participate in the ReSPonD trial. Of the 5 who did not subsequently enroll in the drug trial, *n* = 1 declined, *n* = 2 had likely PDD, and 2 were unable to walk 18 m without an aid. All 5 however participated in the TYM study. Participant recruitment is shown in Figure 1.

The characteristics of our cohort are summarised in Table 1. Participants were predominantly Caucasian with a mean (SD) age of 70 (8.1) years. The median MoCA score was 25 and median TYM score was 43.

### 3.2. Test Distributions

MoCA and TYM assessments were performed on all 135 participants. MoCA scores ranged from 7 to 30 and TYM ranged from 15 to 50. Both measures were negatively skewed. Using the published screening cutoffs for MoCA [11], *n* = 25 (19%) had deficits consistent with PDD, *n* = 49 (36%) had deficits consistent with MCI, and *n* = 61 (45%) had normal cognition. The median time taken to complete TYM was 6.53 mins (interquartile range 5.33 to 8.52 mins). 47% (*n* = 63) of patients required some degree of assistance to complete the test.

### 3.3. Translation between MoCA and TYM

**Figure 2:**
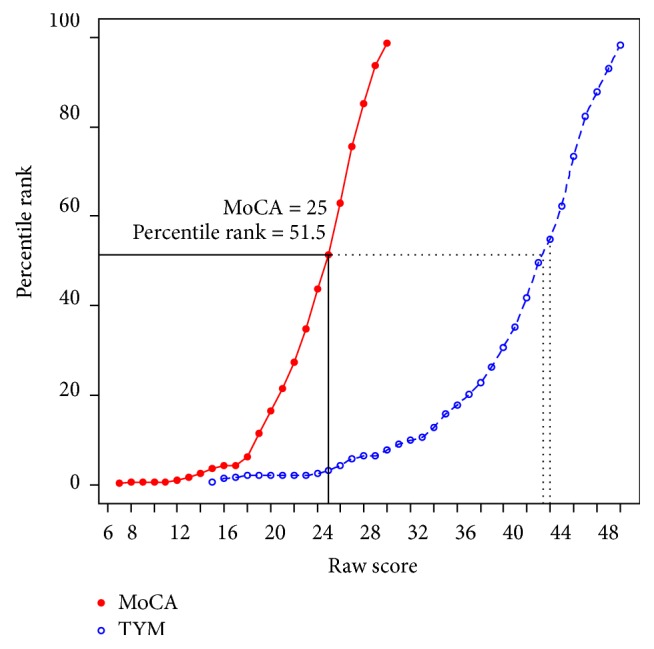
Corresponding raw scores and percentile rank for TYM and MoCA.

Corresponding MoCA and TYM scores, after log-linear smoothed equipercentile equating, are shown in Figure 2 and Table 2. Extrapolated data are shown in italics in Table 2 corresponding to a TYM score of <15. A MoCA score of 25 (the upper limit for screening PD-MCI) corresponds to a TYM score of 43-44, highlighted in bold.

### 3.4. Sensitivity and Specificity of TYM

**Figure 3:**
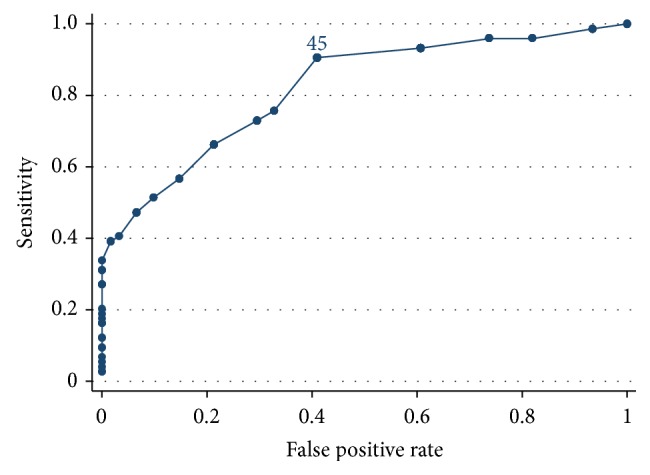
ROC curve showing the sensitivity and specificity of different TYM scores for PD-normal (MoCA 26–30) or PD-cognitive impairment (MoCA ≤ 25). Labelled data point (TYM = 45) gives optimum sensitivity and specificity.

The area under the ROC curve (Figure 3) for TYM to differentiate between PD-normal impairment and PD-cognitive impairment as defined by MoCA was 0.82 (95% CI 0.75 to 0.89). The maximised Youden's *J* statistic with sensitivity 90.5% and specificity 59.0% giving optimum accuracy was a TYM score of 45, which correctly classified 76.3% of patients with PD-cognitive impairment.

### 3.5. Interrater Reliability

The ICC for absolute agreement (ICC = 0.97, 95% CI 0.94 to 0.99, *p* < 0.001) was high, indicating excellent scoring reliability. The median (IQR) difference between the gold standard rater (VL) and the other raters was −1 (−2 to 0) in both cases.

## 4. Conclusions

MoCA is established and advocated as a screening test for cognitive deficits in PD. The main advantage of the TYM test over MoCA and other screening tests is that it is self-administered, being supervised by nonclinical staff, and can be completed whilst waiting at clinic before seeing a specialist. We have established equivalent scores for TYM with MoCA and assessed the discriminant ability of TYM to detect cognitive deficits in PD. Our results suggest that a TYM score of ≤45 identifies MCI level cognitive deficits with a sensitivity of 90.5% and specificity of 59.0%. The relatively low specificity is appropriate for the TYM test's role as a screening test. Using TYM can avoid the need for further testing in many patients; those below the cutoff can be assessed further with MoCA or other tools. If motor problems such as severe tremor affect completion of the writing and drawing tasks, TYM can be completed by another individual under direction from the patient. Neither TYM nor MoCA showed notable floor or ceiling effects in this population. The time taken to complete the test was acceptable [12] and comparable to previously published data [16] even in this PD population. This is the first study that we are aware of that has examined the utility of TYM in PD. In contrast to a previous study [10], a substantial proportion of this cohort (55%) screened positive for cognitive deficits using the MoCA. Despite excluding those with known PDD, our participants had a broad range of cognitive dysfunction severity, which enhances the generalisability of the results.

This study has several limitations. We excluded people with previously diagnosed PDD as they were not eligible to take part in the drug trial in which this substudy was nested. With fewer people with very pronounced cognitive deficits, equivalent scores in the lower range should be interpreted cautiously and this may influence the generalisability of the results. However, we feel that our population of PD patients without dementia but with falls (which are associated with cognitive impairment) represents the group in whom screening for deficits is of most clinical value. We have not compared the TYM to a “gold-standard” test for PD-MCI [23] and PDD [18], but rather to another screening test (albeit one recommended in the diagnostic criteria [level 1] set out by the MDS) [12]. Published MoCA cutoff values for PD-MCI vary slightly between studies. We used a MoCA cutoff score of 26 and may therefore have slightly overestimated those with cognitive impairment. We did not measure the time taken to complete MoCA as a comparison. Although TYM completion took less than 7 minutes, it is probable that people with more severe cognitive deficits would have taken longer.

We would still recommend using the MoCA if concerns are raised regarding cognition as this is the recommended standard validated cognitive screening test in PD [12], which stands alone as a minimum assessment, takes <15 minutes to complete, measures major cognitive domains, and can identify subtle cognitive impairment. Observation of the completion of a cognitive test may afford a clinician further insight into the cognitive changes. Our results suggest that the TYM also meets these criteria, may be faster, and, as it does not require specialist supervision, could further support detection of cognitive deficits in PD. Accurate identification of individuals who require further cognitive assessment is a necessary component of both research testing and clinical testing. Where clinical resource limitations preclude the use of the MoCA, use of the TYM test in PD may be a valuable tool.

## Figures and Tables

**Table 1:** Baseline demographic data.

	Participants (*n* = 135)
Mean age	70.0 (8.1)
Sex (*n* female (%))	51 (38%)
Caucasian ethnicity	134 (99%)
Age at leaving school	16 (15–17)
Montreal cognitive assessment (total score)	25 (22–27)
“PD-normal impairment” (MoCA 26–30)	61 (45%)
“PD-cognitive impairment” (MoCA ≤ 25)	74 (55%)
Test your memory (total score)	43 (39–46)
Total MDS-UPDRS (total score)	90 (74–106)^*∗*^
Duration of PD (yrs)	9 (5–13)^*∗*^

^*∗*^ *n* = 130.

**Table 2:** Equivalent TYM and MoCA scores.

TYM	MoCA	TYM	MoCA
*0*	*0*	25	15
*1*	*1*	26	16
*2*	*2*	27	16
*3*	*3*	28	17
*4*	*4*	29	17
*5*	*5*	30	18
*6*	*6*	31	18
*7*	*7*	32	19
*8*	*7*	33	19
*9*	*8*	34	20
*10*	*8*	35	20
*11*	*9*	36	21
*12*	*9*	37	21
*13*	*10*	38	22
*14*	*10*	39	22
15	11	40	23
16	11	41	23
17	12	42	24
18	12	**43**	**25**
19	12	44	25
20	13	45	26
21	13	46	27
22	14	47	27
23	14	48	28
24	15	49	29
25	15	50	30

Italics indicate extrapolated data.
